# Making tacit knowledge explicit through objects: a qualitative study of the translation of resilience into practice

**DOI:** 10.3389/fpubh.2023.1173483

**Published:** 2023-06-26

**Authors:** Hilda Bø Lyng, Cecilie Haraldseid-Driftland, Veslemøy Guise, Eline Ree, Heidi Dombestein, Birte Fagerdal, Hilde Valen Wæhle, Siri Wiig

**Affiliations:** ^1^SHARE–Centre for Resilience in Healthcare, Faculty of Health Sciences, University of Stavanger, Stavanger, Norway; ^2^Department of Research and Development, Haukeland University Hospital, Bergen, Norway

**Keywords:** boundary objects, epistemic objects, activity objects, resilience in healthcare, learning tool, interventions

## Abstract

**Introduction:**

It is common practice to use objects to bridge disciplines and develop shared understanding across knowledge boundaries. Objects for knowledge mediation provide a point of reference which allows for the translation of abstract concepts into more externalized representations. This study reports from an intervention that introduced an unfamiliar resilience perspective in healthcare, through the use of a resilience in healthcare (RiH) learning tool. The aim of this paper is to explore how a RiH learning tool may be used as an object for introduction and translation of a new perspective across different healthcare settings.

**Methods:**

This study is based on empirical observational data, collected throughout an intervention to test a RiH learning tool, developed as part of the Resilience in Healthcare (RiH) program. The intervention took place between September 2022 and January 2023. The intervention was tested in 20 different healthcare units, including hospitals, nursing homes and home care services. A total of 15 workshops were carried out, including 39-41 participants in each workshop round. Throughout the intervention, data was gathered in all 15 workshops at the different organizational sites. Observation notes from each workshop make up the data set for this study. The data was analyzed using an inductive thematic analysis approach.

**Results and conclusion:**

The RiH learning tool served as different forms of objects during the introduction of the unfamiliar resilience perspective for healthcare professionals. It provided a means to develop shared reflection, understanding, focus, and language for the different disciplines and settings involved. The resilience tool acted as a boundary object for the development of shared understanding and language, as an epistemic object for the development of shared focus and as an activity object within the shared reflection sessions. Enabling factors for the internalization of the unfamiliar resilience perspective were to provide active facilitation of the workshops, repeated explanation of unfamiliar concepts, provide relatedness to own context, and promote psychological safety in the workshops. Overall, observations from the testing of the RiH learning tool showed how these different objects were crucial in making tacit knowledge explicit, which is key to improve service quality and promote learning processes in healthcare.

## 1. Introduction

It is common practice to use objects to bridge disciplines and develop shared understanding across knowledge boundaries (1). Research-based interventions for quality improvement in healthcare settings require joint efforts and involvement of multiple stakeholders with different backgrounds (2). Often therefore, quality improvement efforts require translation of research into practice and the mediation of knowledge across the different stakeholders involved.

Boundary objects have been found valuable for mediating knowledge in the implementation of quality improvement interventions in healthcare (3). A boundary object is something that provides a point of reference which allows for the translation of meaning and the transformation of abstract concepts into more concrete representations (1, 4, 5). Boundary objects may take many different forms. Concrete representations, like prototypes, are frequently described in research (1, 4, 6), but also metaphors and analogies (5, 7), visual representations (8, 9), narratives (10), and processes and methods (11) have proved useful as objects for knowledge mediation. Boundary objects are not permanent entities, hence they can adapt and transform in terms of their role, function and setting throughout a project (1, 5).

Based on the diverse role and function occupied by boundary objects in different settings, criticism has been raised regarding the “one size fits all” use of the boundary object term. Nicolini, Mengis (1) claims that different forms of objects have been described and used in research and one should therefore be more specific in the way one uses terms. They further separate objects for collaboration and knowledge mediation into boundary objects, epistemic objects, activity objects, and infrastructure objects. Boundary objects through their translation and transformation abilities provide means for making collaboration across different disciplines, organizations, and system levels possible. Epistemic objects are open ended objects that embody what is yet to be known and are shown to provide motivation and engagement in collaborations (1, 5). In this way, boundary objects make collaboration viable (providing shared understanding), while epistemic objects provide us with understanding of *why* people collaborate (motivation to collaborate) (1). Activity objects also provide motivation for collaboration, but unlike epistemic objects, activity objects do so through collective actions (1, 12, 13). Furthermore, infrastructure objects refer to objects that support the implementation of collaborative work (1).

Despite the recognized value of mediating objects for collaborations, benefits gained by the objects cannot be determined beforehand due to the context specific nature of mediating objects. Objects are not magical solutions, and even thoughtfully developed objects may fail to provide mediating value (4, 6, 14), meaning that the values, roles and functions of objects need to be studied in different contextual settings. Furthermore, objects are not static entities, instead objects align to a life cycle nature where the impact changes throughout the collaborative process. This implies that objects may take different roles in accordance with different phases of the process (15).

Interventions designed to improve healthcare quality introduce changes, either in the form of new ways of acting or through new ways of thinking. The adoption of new practices and perspectives is challenging as it questions our cultural and contextual knowledge (14, 16). The way we do things is often founded on tacit knowledge, which is not easily transferable across knowledge boundaries (16–19). The externalization of tacit knowledge, like codifying ‘work-as-done’ in healthcare (processes, procedures, perspectives), has been found difficult and even when performed, it may still be incomprehensible if the externalization is not provided in a form that creates a shared understanding (14, 20). Quality improvement interventions therefore need means for externalization of knowledge and for the development of shared understanding, in which boundary objects have been described as valuable (21).

### 1.1. The resilience in healthcare research program

This study is part of the Resilience in Healthcare (RiH) research program (22, 23), which aims to establish a comprehensive RiH framework for identifying and strengthening resilience in healthcare. In this research program, resilience in healthcare is defined as: *“the capacity to adapt to challenges and changes at different system levels to maintain high quality care,”* and as such focus is directed toward adaptive capacity, learning from what goes right, and to provide understanding of everyday practices (24), p. 6. The main objectives of the RiH program are to translate and operationalize the RiH perspective into practice, and furthermore to provide healthcare professionals with a research-based tool for learning about resilience and what leads to positive outcomes in their clinical context. In further support of the operationalization of resilience in healthcare, previous studies in the RiH program have identified 10 capacities for resilient performance across diverse healthcare settings (25). The resilience capacities include structure, learning, alignment, coordination, leadership, risk awareness, involvement, competence, facilitators, and communication, and they form the empirical and theoretical basis for developing the content of the RiH tool to translate resilience into practice.

In the Norwegian setting, resilience in healthcare is perceived as a new and unfamiliar perspective on healthcare quality. The traditional way of thinking about quality and patient safety largely entails learning from mistakes, adverse events, and near misses. The introduction to practice of a RiH tool, which promotes the aim of learning from success, therefore requires translation into practice, alongside the development of a shared understanding and learning of a new way of thinking. This article reports findings from the initial testing of a tool developed to translate resilience into practice and investigates how we can understand the role of objects in this process.

The development process of the RiH learning tool was based on the approach of ‘perspective taking’, where new knowledge is to be “exchanged, evaluated, and integrated with that of the others in the organization” as a way to introduce a new perspective (8), p. 358. However, in what ways the RiH learning tool would provide translation, operationalization and learning regarding the resilience perspective was uncertain, and was therefore of particular interest in this study.

### 1.2. Aim and research question

The aim of this paper is to explore how a RiH learning tool may be used as an object to introduce and translate a new quality and safety perspective into practice across different healthcare settings.

The research questions are:

How does a RiH learning tool act as a mediating object for the translation of resilience in healthcare?

What contributions does a RiH learning tool make to the introduction of a novel perspective of quality improvement in healthcare?

## 2. Methods

### 2.1. Study design

We report and discuss findings from the test phase of the RiH project (22, 23), where we tested a newly developed resilience in healthcare (RiH) learning tool in different healthcare contexts. The purpose of the RiH learning tool is to translate the resilience in healthcare perspective into practice; change the way safety is approached and perceived in practice; learn from what goes well in clinical settings; and to promote adoption of a systems resilience perspective among healthcare professionals. To allow for in-depth exploration of this phenomenon a qualitative explorative research design was conducted. This included focus group interviews prior to and after the introduction and test of the tool, as well as observation of intervention activities during the test phase. This article reports findings from the observations only, in an effort to explore the role of the RiH learning tool as a mediating object of translating knowledge into practice.

### 2.2. Description of tool

The RiH learning tool is designed to assist diverse healthcare units, such as teams, wards, or organizations, to understand the resilience perspective, what resilience capacities are, and to enable identification of patterns of own resilient performance through collaborative assessment and discussion of everyday practice. The tool is made up of three different elements to allow for flexible use within dynamic healthcare settings; Element 1 (Mapping tool), Element 2 (Learning scenarios) and Element 3 (Reflection tool). Each element can be used separately or as part of a step-by-step process moving from one element to the next. The development of the RiH learning tool elements in terms of technological features, aim, content, and approach is described in Table 1 (19).

**Table 1:** Description of RiH learning tool.

Elements	1. Mapping	2. Learning scenario	3. Resilience reflection
Aim	Provide an overview of status of own unit related to resilience capacities	Provide understanding of resilient capacities and how the unit solve situations in a good way	Continued focus and adoption of resilience capacities and perspective
Content	Instruction guidelines	Instruction guidelines	Instruction guidelines
	Descriptions of resilience capacities	10 unique practice- based narratives	Resilience reflection list
	30 different statements related to the resilience capacities	60 different learning scenario elements.	Descriptions of resilience capacities
		110 reflective questions	
Approach	Collaborative learning approach where groups of healthcare personnel work together	Collaborative learning approach where groups of healthcare personnel work together	Collaborative learning approach where groups of healthcare personnel work together
Technical features	Short e-learning videos	Instructional e-learning videos	Printable pocket card
	Digital questionnaire with Likert scale	Video narratives containing pictures, voiceover and subtitles.	Handouts: Pocket card in waterproof material for easy disinfection
	Interactive wheel describing the 10 different capacities		Interactive wheel describing the 10 different capacities.
	Sector diagram visualizing results based on learning algorithms		

Facilitation to understand the resilience perspective was in this tool constructed by including three e-learning videos; one introductory video, one as part of the Mapping tool (Element 1) and one as part of the Learning scenarios tool (Element 2), as well as through descriptions of the 10 resilience capacities as part of the Mapping tool (Element 1) and the Reflection tool (Element 3), see Table 1. Discussion of everyday practice was constructed through the inclusion of practice-near statements in the Mapping tool (Element 1) and reflexive questions in the Learning scenarios tool (Element 2) and in the Reflection tool (Element 3). Semantically framed to help the participants relate resilience to their own setting. Collaborative learning was constructed in all elements by encouraging the participants to discuss and negotiate joint responses to both the practice-near statements and reflexive questions and then recording these joint responses in the learning tool. Furthermore, feedback and results from the mapping of their own unit as part of the Mapping tool (Element 1) facilitated participants’ learning of resilience, see Table 1.

### 2.3. Data collection, setting and participants

**Figure 1:**
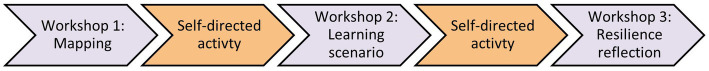
Overview of intervention design.

The study findings are based on empirical observational data. The intervention to test the tool took place from September 2022 to January 2023. The intervention entailed three, sequential researcher-led workshops where representatives from each unit received training in how to use the different elements of the RiH learning tool. In between each researcher-led workshop the unit representatives were instructed to undertake specific self-directed activities in their respective unit, before returning to the next researcher-led workshop. The topics of the three workshops (WS) were: WS1; Mapping, WS2; Learning scenarios, and WS3; Resilience reflection. See Figure 1 for an overview of the intervention design, which displays the topics and sequence of workshops and self-directed activities. All three workshops consisted of parts A and B. In part A the participants received information related to the background and intent of an element of the tool and instructions for use, while in part B they tested the learning tool for themselves and planned how to conduct the upcoming self-directed activity in their own unit. In workshops two and three, part A also entailed discussions of how the previous self-directed activity had been accomplished, alongside positive and negative aspects of the use and content of the tool and its impact on the unit.

Throughout the intervention, data was gathered through observations conducted at each of the three workshops at all the intervention sites. Observations were only undertaken at the researcher-led workshops, and not during the self-directed learning activities. The intervention program was run as one intervention at each site, meaning that all units at the same site participated in the same set of three workshops. This was intentionally set up to facilitate learning between units from the same organization. A total of 15 workshops were carried out, at five sites including hospitals, nursing homes and home care services (see Table 2). These five sites included a total of 20 units (long-term and short-term nursing home wards, home care services, hospital quality departments, medical wards, and outpatient units) and between 39 and 41 participants in each of the three workshop rounds. The recruitment of sites and participants was initially done through the researchers’ contacts at the different sites, before securing formal approval from the management at each site. The workshop participants were responsible for including additional staff from their unit to participate in the self-directed activities between workshops. How this was carried out in practice varied from site to site, from unit to unit, and from the first self-directed activity to the second. Some of the units only involved one or two additional participants in the self-directed activities while others involved all staff in their unit, ranging between 15 and 25 participants. No patients were involved in the intervention process or data collection. During the intervention period, the Norwegian healthcare system suffered additional strains related to a high level of covid related deaths, as well as reports of workforce burnout and increased levels of turnover and sick leave. All sites and units involved in the study reported issues related to these challenges and several of the participants therefore only took part in some parts of the intervention.

**Table 2:** Overview of sites, units, and number of participants in workshops.

Site number	Number of units involved	Number of participants from all involved units	Setting
1	1	2	Home health care service
2	2	2	Nursing home
3	4	11	Hospital
4	10	18–20 (varied across workshops)	Nursing home
5	3	6	Hospital
Total	20	39–41	

Pairs of researchers were responsible for carrying out interviews, observations, training, documentation of observations, and providing feedback at each of the five sites. Some of the researchers were involved at several sites, while other researchers were only involved at one site.

### 2.4. Data material and analysis

Observation notes from all 15 workshops make up the data set for this study, based on approximately 30 h of observation. All observation notes were written up after the workshops in accordance with a predefined observation template, which included the following main themes and subthemes: introduction of participants, experiences from the self-directed activities with the tool in their own organization, usability and functionality of the tool, training of new procedures, interaction between participants, reflection over resilience concepts, and the researchers’ own post-workshop reflections. All researchers took part in workshops, observations, and the writing up of observation notes.

**Figure 2:**
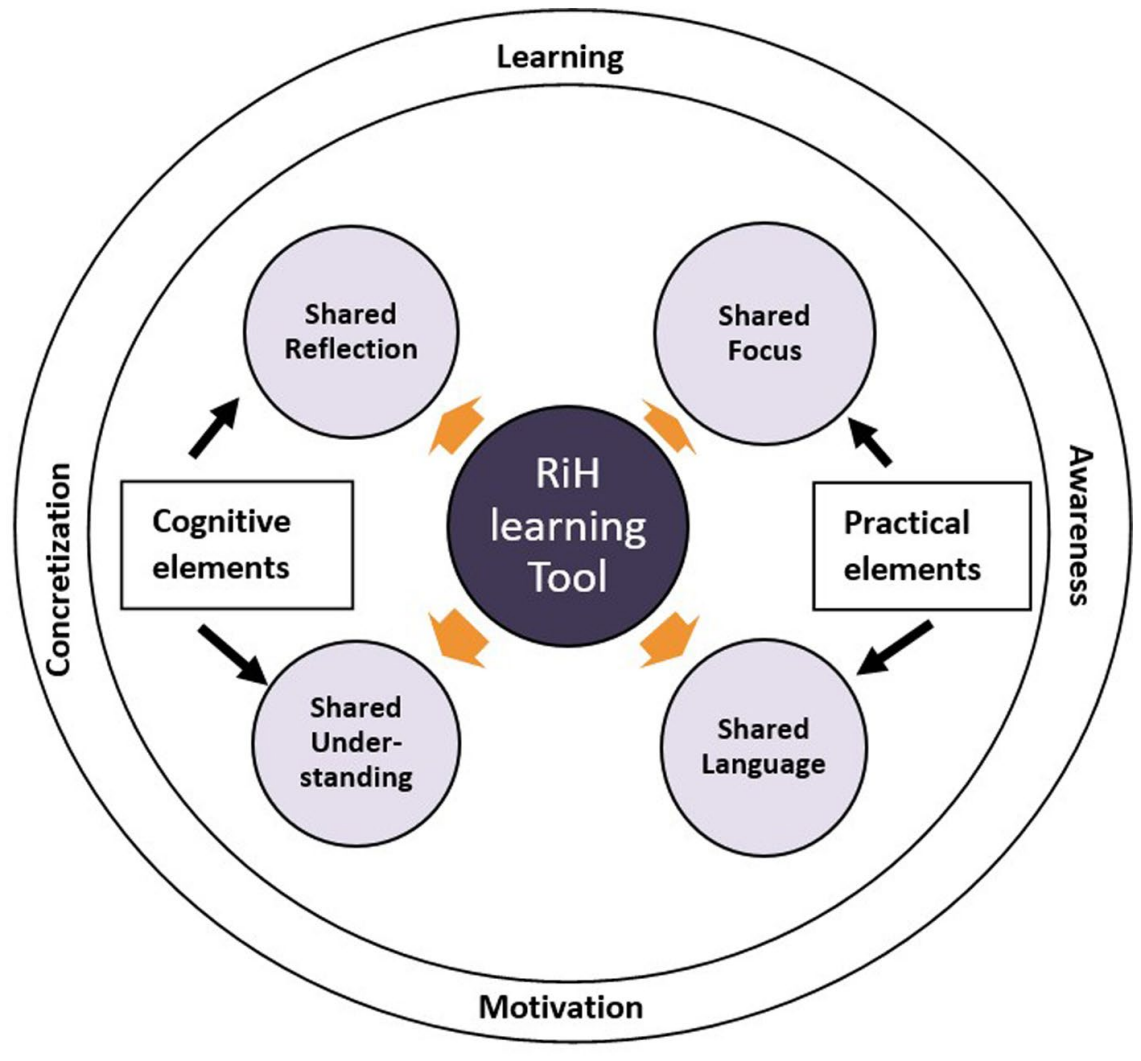
RiH learning tool as a mediating object.

**Figure 3:**
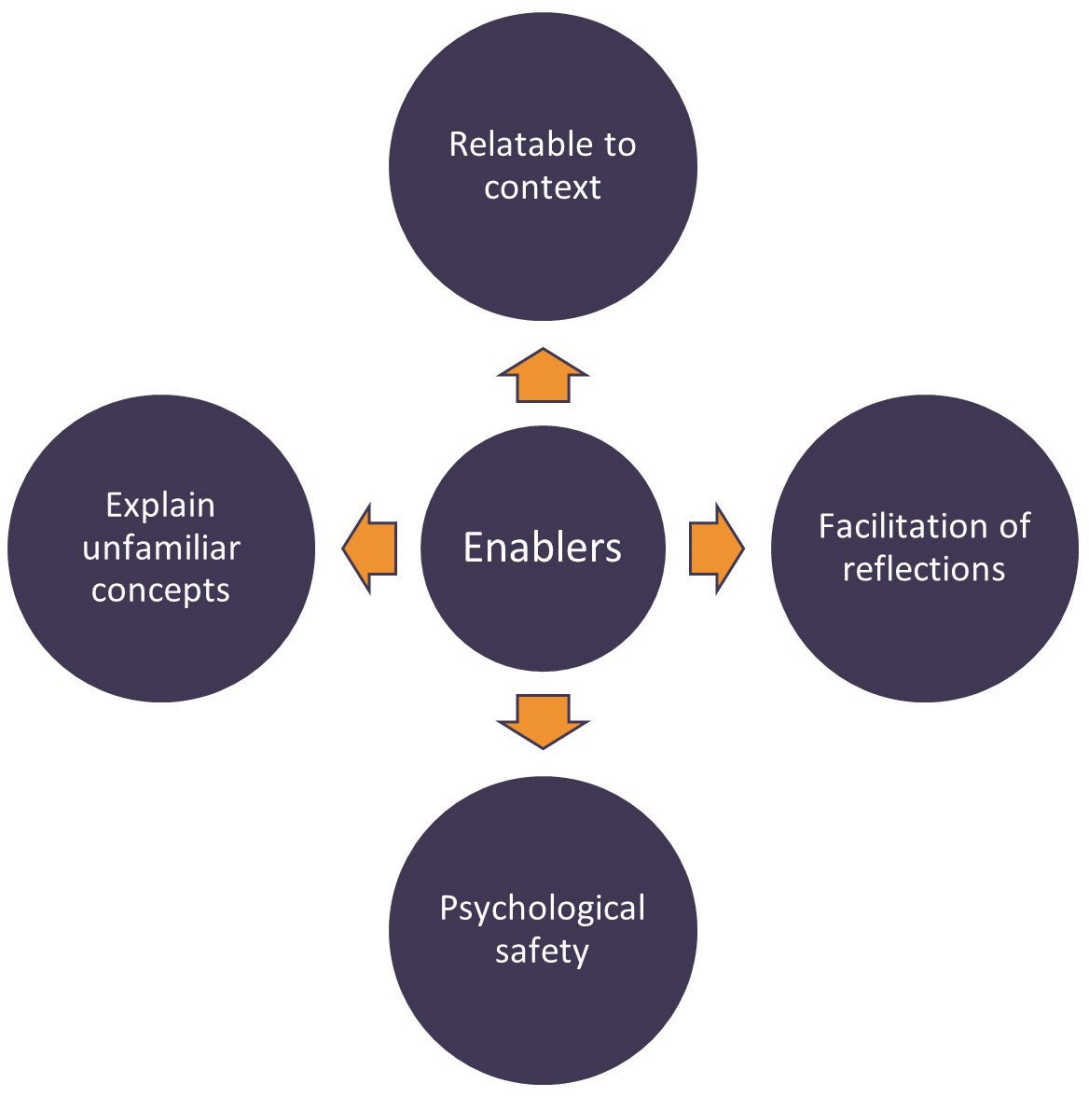
Enablers for translating resilience into practice.

Data were analyzed by way of a thematic analysis approach inspired by Braun and Clarke (26), and followed an inductive approach. The analytical process included five phases. First, all observation notes were imported in the template and read by all authors, who all had been involved in the facilitation of the workshops. Second, author HBL coded initial sub-themes emerging directly from the text. Third, the sub-themes were discussed within the researcher group to agree on which sub-themes were of relevance for this study and further aggregation into themes. Fourth, the researcher group discussed the synthesizing of the themes into those illustrated in Figure 2 (initially proposed by HBL and CHD) and agreed that this figure represent a visual display of our findings. Furthermore, the fourth phase identified the relatedness in role and function between our findings and the meditation object literature to distinguish which type of objects the findings correspond to. Fifth, the themes were revised, and some wordings of themes were slightly changed, e.g., shared goals were renamed shared focus. Furthermore, the researchers discussed the relationships illustrated in Figure 3, which was developed and proposed by author HBL. All researchers involved in this study are experts in qualitative methods and familiar with interventions, observation studies, and thematic analysis.

## 3. Result

Findings from this intervention to test a RiH learning tool in diverse clinical settings show that resilience as a perspective for understanding work-as-done, quality of care and patient safety was a highly unfamiliar approach among workshop participants. Participants have been used to the traditional emphasis within the Norwegian healthcare system on learning from adverse events (also known as the safety-I perspective). Outcomes from the introduction of a new quality and safety perspective in clinical practice will be presented below, focusing on how the RiH learning tool functions as a mediating object to translate novel ideas into practice.

### 3.1. Rih learning tool as a mediating object

The RiH learning tool acted as a mediating object through the provision of shared reflection and shared understanding, categorized as cognitive elements, for the different actors. Furthermore, the RiH learning tool contributed to a shared language and shared focus for the involved participants in terms of elements related to practice. The overall contribution of the RiH learning tool as a mediating object for different healthcare disciplines and roles was in its facilitation of learning, motivation, awareness, and concretization. Results from the inductive analysis are depicted in the model in Figure 2, while examples of the inductive empirical themes can be found in Table 3.

**Table 3:** Themes and related examples from workshop observations.

Themes	Empirical example
RiH learning tool as a mediating object	
Shared reflection	During the workshop with a total timeframe of 1,5–2 h, 15–30 min were used to introduce the next element of the tool and the process required to undertake the ‘homework’ (train-the-trainer approach). The rest of the time was dedicated to reflections. We observed great engagement in the reflections. And the participants needed to be paused at times to be able to move on. After the workshop ends, we observed participants continuing with reflections.
Shared understanding	The participants declare that they were surprised at the level of consensus, during mapping and prioritization activities, across organizational levels
Shared language	Participants state that they were able to read and understand the statements in the mapping tool and to use this wording when describing their own situation
Shared focus	The resilience perspective, and the tools, provide a way of developing a common focus on what they are good at in groups, thus forming cross-disciplinary, cross-organizational, and cross-level agreements.
Enablers for translating resilience to practice	
Easy relatable to context	The participants use practical examples when reflecting on the resilience concept and the capacities, e.g., for alignment, the participants tell us that they align their work to changing conditions throughout the workday. They, for instance, need to welcome new patients with unfamiliar problems and accompanying unfamiliar procedures, and align their work when staff call in sick.
Facilitation of reflections	The facilitator needs to make sure that participants reflect upon what goes well in their practice instead of discussing what they need to improve.
Psychological safety	The facilitator succeeds in creating psychological safety. All participants contribute to the reflection and report the feeling of a safe and informal environment during the workshop
Explain unfamiliar concepts	The participants report that the clarification of concepts makes them able to put their own work practice into words

#### 3.1.1. Shared reflection

Having an arena to engage in reflection over what works well in their practice was appreciated by all participants. Due to the unfamiliarity of the resilience perspective, explaining resilience and the associated resilience capacities was needed as a starting point to allow for subsequent shared reflection. Once concepts had been clarified, participants engaged eagerly in the reflection rounds, to the extent that these had to be paused at points to move forward. The engagement in reflection was not found to be restricted to the workshop, as we observed prolonged reflection after the workshops ended which also became evident at the next workshop. The resilience perspective and the resilience capacities were found easily translatable to participants’ work practices and settings, although it did demand an initial familiarization with the concepts.

Some sites reported that having a combination of newly hired personnel together with experienced personnel as very rewarding during the reflection. They reported that new staff members more easily could discover and value what worked well in their setting. Furthermore, participants experienced that both experienced and new personnel could *learn* from each other during the reflections. Usually, finding time for knowledge transfer during their daily hectic work schedules was difficult but their engagement with the RiH learning tool supported knowledge exchange processes.

#### 3.1.2. Shared understanding

We observed that the tool provided a shared understanding of important elements of resilience, quality of care and patient safety. The level of *concretization* in participants’ reflections improved in proportion with their shared understanding of the concepts. Thus, the explanatory text included on the website and within the different elements of the tool, as well as the time set aside for clarification of concepts in every workshop, provided a shared understanding of the resilience perspective which again improved reflection. Some participants reported surprise at the high level of consensus, across organizational levels (leaders and healthcare workers), happening in the reflections. Others stressed the value of reflecting and negotiating with people who have a different perspective than your own. Due to the practice-near nature of the RiH learning tool, the different participants related the resilience perspective to different aspects of their situated work. As such, the participants brought different operationalizations of resilience in practice to the discussion. However, as the resilience capacities are of a quite generic nature, these differences were not found to hamper the development of a shared understanding. Since the tool elements were designed in such a way that the participants need to reach a consensus before reporting a joint response, the tool prompts the need for participants to agree upon a shared understanding during reflections. For the participants in this intervention, this was sometimes a straightforward process, while at other times it relied on converging reflections within a group of colleagues. However, these discussions were found to provide valuable reflections in the group work due to their differing work-related operationalizations. The practice-near foundation of the RiH learning tool meant that some aspects of the scenarios were found to be more easily relatable for healthcare professionals working directly with patients, as opposed to those in managerial and administrative positions.

#### 3.1.3. Shared focus

The resilience perspective was found to be unfamiliar to all participants. This unfamiliarity introduced a challenge for the researchers in terms of keeping workshop reflections and discussions within the resilience perspective.

When reflections centered around a shared focus on resilience and what works well, participants reported this shift in focus to provide *motivation*, which was highly needed in the post-covid healthcare context. The new focus on positive experiences was therefore highly welcomed by the participants. Reflections on what they are good at and what works well in their everyday clinical work provided a shared focus leading to the development of shared goals. However, a change of focus to an unfamiliar methodology required structured efforts from facilitators, repeated explanations and clarifications, and a willingness to embrace new ways of thinking. To keep a shared focus over time, several sites decided to do the work with the self-directed activities as part of already planned meetings in their units. By doing so they worked on small parts of the tool over a longer time period. An example was where one unit incorporated reflection on one or two statements from the first element of the tool during their routine morning meeting over the course of several days.

#### 3.1.4. Shared language

Working with the different elements of the tool allowed for rich reflections across disciplines, roles, and organizational boundaries. A highly important outcome of these reflections was the development of a shared language to concretize what works well. Through the shared language provided by the tool, participants got an *awareness* of which of the capacities that contributed to well-functioning work practices. We observed a marked improvement in how they described their work from the first to the third workshop. For example, they went from saying “we interact well with family members of patients” which only provides general knowledge, to saying “we have good structures in place for the involvement of next-of-kin, like monthly updates via phone calls,” which provides *concretization* of what works well to facilitate involvement. Concretization allows for best practices to be transferred to other teams and organizations, thereby encouraging broad *learning* from success.

### 3.2. Enablers for translating resilience to practice

The rewards gained by translating the resilience perspective to practice is not as straight forward as just giving healthcare providers a resilience tool. The translation of the resilience perspective to practice was found in this study to rely on several enabling factors. Enabling factors observed in the workshops were facilitation of reflections, to establish psychological safety, to explain unfamiliar concepts, and to provide relatedness to a specific context. See Figure 3 for an illustration of these enablers for translating resilience into practice.

#### 3.2.1. Facilitation of reflections

The RiH learning tool intervention relies on a ‘train-the-trainer’ methodology. The workshop participants were therefore expected to go back to their own unit/organization to facilitate several self-directed activities together with their colleagues. It became apparent that achieving fruitful reflections, where all participants contribute and have the same focus, need targeted facilitation (both in the workshops and in self-directed activities). As the researchers listened in on the reflections taking place during the researcher-led workshops, there was a noticeable tendency for participants to drop back into a familiar quality and safety perspective with discussions of areas in need of improvement. Facilitators, therefore, had to interact with and guide participants to keep reflections within the resilience perspective, thereby encouraging them to focus on positive outcomes when discussing everyday practice within their own units.

#### 3.2.2. Psychological safety

Another enabling factor to encourage good reflections was to establish an atmosphere characterized by psychological safety. This was achieved by researchers engaging in informal conversation and small talk with the participants, asking questions about their work and daily life, and by complimenting and encouraging their contributions to the conversation. The facilitators also supported the participants whenever they expressed difficulties in focusing on positive, rather than negative outcomes, thereby attempting to take the stress out of dealing with learning a new approach to quality and safety work. The participants reported the workshops to be informal, respectful, and welcoming.

#### 3.2.3. Explain unfamiliar concepts

Introducing an unfamiliar perspective on healthcare quality and safety posed a need for facilitators to explain concepts and the background and rationale for using a resilience approach in the participants’ more familiar disciplinary language. To do so the facilitators needed some knowledge and understanding of the participants’ contextual settings, to provide them with practice-based examples and terms. In doing so, the facilitators took on the role as boundary spanners (bridging two disciplines, e.g., nurse and researcher) (27). Despite providing information to the participants prior to the start of the workshops, the resilience concept was perceived as foreign until it had been explained as part of the first workshop using concrete examples from the healthcare context. Furthermore, the unpacking of the resilience concept continued beyond the first workshop and remained a point of discussion and reflection across all three workshops.

#### 3.2.4. Relatable to context

The need for participants to relate the resilience perspective to their own context and practice was found crucial for operationalization and concretization. As such, the facilitator continuously requested practical examples from participants during the reflection sessions. A marked intention was to ensure that the participants kept discussing work-as-done by asking questions like “how do you do this in your team/organization?,” instead of reflections of work as imagined, with participants talking about what they ought to be doing or wish they could be doing, for example “we wish we had meeting arenas to talk about these things.” Keeping the focus on work as actually done in the real and explicit context of participants was thus an issue in need of continuous attention from the facilitators of the workshops.

## 4. Discussion and conclusion

In this study we have explored the role of objects in a novel effort to translate resilience into practice when implementing a RiH learning tool in Norwegian nursing homes, homecare, and hospital units. In response to the call for specificity in the way objects act in collaborations (1), the following discussion will debate the different forms and functions of the RiH learning tool throughout the intervention process, see Table 4, which echoes the understanding of objects being able to change form and function (1). The RiH learning tool was found to act as three different objects described in the literature, namely boundary objects, epistemic objects, and activity objects.

**Table 4:** How the RiH learning tool acted as boundary objects, epistemic objects, and activity objects for translating the resilience perspective into practice.

Function	**Shared language**	**Shared understanding**	**Shared focus**	**Shared reflection**
Form	Boundary object	Boundary object	Epistemic object	Activity object
Type of enabler	Explain unfamiliar concepts	Relatable to context	Facilitation of reflection	Psychological safety
Operationalized in intervention	The resilience perspective and all concepts are explained in the intervention, both in text and in videos	The tool elements (Mapping and Scenarios) direct participants to relate their reflections to their own context: e.g. “How is this in your team”	The tool elements are designed to focus participants on the resilience perspective. For example, in the Scenarios element, reflections are related to successful clinical scenarios displayed in videos. Participants reflect on why and how things went well in the scenarios and how similar situations would act out in their own contexts	The tool elements are based on a collaborative approach, where participants work in groups with the different tools

### 4.1. Boundary objects

The RiH learning tool acted as a boundary object in providing shared understanding and a shared language across disciplines and diverse healthcare settings. Unfamiliarity with the resilience perspective made it necessary for participants to have objects for the translation of unfamiliar concepts and perspectives to their situated practice (21). For the RiH learning tool, translation and transformation was provided by designing the tool elements to direct participants to relate concepts to their own context. Knowledge conveyance and knowledge convergence provide ownership and internalization of new and unfamiliar perspectives (28, 29). Knowledge conveyance includes the transfer of an appropriate amount of new knowledge, here provided through explanations of resilience concepts and terms, both verbally (in workshop presentations and in scenario videos) and as part of the written material included in the tool. Knowledge convergence refers to the convergence of different perspectives, here introduced by converging new knowledge with traditional perspectives of care quality and patient safety.

Actions of knowledge conveyance and convergence need to be repeated in a cyclic manner (29). This is echoed in our study where the unfamiliarity of the introduced resilience perspective called for repeated explanations of resilience and its contribution to the specific healthcare services included in the study. As the intervention progressed the development of a shared language became apparent, and the participants were able to concretize the way they spoke about their own context, practices, quality of care and resilient performance. As such, this study contributes new understanding of how a resilience tool, acting as a boundary object, provided a way of externalizing tacit knowledge. Through the development of a shared language the participants were able to put tacit knowledge of how they do their work, including adaptations, workarounds, and what works well, into words. This externalization further provided a way for sharing experiences and best practices across teams, wards, and organizations.

### 4.2. Epistemic object and activity objects creating motivation for improving quality

The RiH learning tool acted as an epistemic object in providing motivation and engagement for the participants through the development of shared goals. Nicolini, Mengis (1) exemplifies epistemic objects as shared goals and research ideas, providing engagement and motivation for further cross- disciplinary collaboration. This is reflected in this study, where a shared focus on resilience and learning from what works well clearly provided the participants with motivation. However, participants found it challenging to keep the focus of reflections within the resilience perspective. Having a facilitator in the reflection sessions was therefore very constructive. The role of the theoretical systemic foundation was essential and echoes previous studies of how the systemic perspective is key in translating theory to practice. Similar to what was reported by Wiig, Robert (3), our study showed that the systemic orientation changed the way participants talked about quality and safety and enabled new perspectives within these groups of healthcare professionals.

Furthermore, the RiH learning tool elements acted as activity objects. The main objective of the RiH learning tool is to ensure reflection sessions for the understanding, ownership, and operationalization of the resilience perspective in diverse healthcare settings. Activity objects are described as “incomplete, emergent, and expansive which gives them their performative character” (1), p. 624. In this study, this was present through the provision of open-ended reflective questions which participants must relate to their own context. Acting with objects, here the RiH learning tool, is described as a sense maker by reflecting the meaning and value of the object (13). This is echoed in our study, where the level of engagement was found considerably higher when working with the learning tool in smaller groups, compared to in large plenary discussions. This further reflects the need for the presence of psychological safety during such activities and the importance of establishing a safe environment for all participants to contribute to the reflection sessions.

Both epistemic objects and activity objects are found to support motivation. Michie, Van Stralen (30) describe how people’s ability to change their behavior relies on capability, opportunity, and motivation. The use of the RiH learning tool was therefore a way for participants to develop motivation to adopt a new perspective in their work, changing their way of thinking and learning. This finding may reflect the value of using a resilience perspective, where one seeks to learn from what goes well instead of only learning from mistakes and adverse events. However, the uptake of new perspectives and knowledge relies on a willingness to be open to change and new ways of thinking, which requires an internal motivation and thus cannot be forced through external motivators (31, 32).

### 4.3. Implications

Findings from this study provide both theoretical and practical implications for similar interventions aimed at introducing new theoretical perspectives to the healthcare setting.

#### 4.3.1. Implications for theory

This study extends the resilience in healthcare literature by providing an empirical understanding of how to translate the resilience perspective into practice. Furthermore, this study also extends the object literature through the provision of an empirical understanding on how a RiH learning tool acted as different forms of objects throughout an intervention to introduce a new quality and safety perspective across different healthcare settings. Calls have been made to provide clarification of different types of objects (1), and this study contributes an understanding of how a RiH learning tool acted as boundary object, epistemic object and activity object during the intervention, see Table 4.

This study reports from the introductory phase of a resilience intervention, where a new resilience perspective was introduced for healthcare professionals in clinical practice. As both resilience and objects are not possessable stable phenomena, but instead dynamic aspects that change over time, the findings in this study cannot determine whether resilient performance was subsequently achieved or adopted at the involved study sites, nor can they determine whether the learning tool would act as an object at later phases of resilience adoption. The understanding contributed by this study is based on the introductory phase only, where a learning tool was found to act as different mediating objects for introducing the resilience perspective to the practice field.

## 5. Implications for practice

This study also contributes with implications for practice. Firstly, the learning tool was found helpful to aid the introduction and translation of a new perspective like resilience in healthcare to practice by encouraging the development of shared focus, understanding, language and reflection. The initial participants reported a lack of meeting arenas where they could engage in reflection within their organizations. Healthcare providers and leaders should therefore consider providing their staff with reflexive spaces to promote this learning and practice improvements. This was highly appreciated by the participants in our study, which is in line with suggestions from the literature on how to leverage resilience into practice (33).

Learning new work practices and perspectives requires time, openness and interest on the part of the learners. The RiH learning tool described here acted as an object that can bring people together with a common purpose. This is of utmost importance in improvement activities. In addition, the built-in flexibility in how to use the tool in diverse healthcare settings was found to be important and implies that future efforts to support resilience in practice need to take this into account. Using tools for knowledge translation and to learn new perspectives requires bringing people together (in real life or digitally), it requires a tool with a solid theoretical foundation, and it needs structured activities and support as part of its introduction into workplaces.

However, setting aside time and resources for engaging in interventions relies on interventions to be anchored in leadership (34, 35). Implementation frameworks for healthcare services interventions emphasize intervention sustainability (36). This is in line with results from a previous leadership intervention in healthcare finding management continuity to be key for engaging in the intervention and for the implementation of quality and safety measures (36), and management anchoring was a key factor for intervention sustainability (37).

Despite the current digitalization trend, findings from this study illustrate the utility of face-to-face facilitation and physical meetings. The study participants needed guidance and repeated explanations to understand the new perspective, as well as active facilitation during reflections. They stressed the value of facilitators being physically present and questioned if they would be able to succeed to the same degree if the intervention workshops had been provided only digitally. The physical presence aspect of facilitation is thus something that needs to be considered when designing future resilience interventions for the healthcare setting.

Some of the workshops reported in this study included participants from several teams and wards within the same organization. An intra-organizational approach was found to be valuable to support learning and the sharing of best practices across differing teams and wards and was appreciated by the participants. Using a cross-organizational approach requires efforts to develop psychological safety within the workshops. Psychological safety was addressed in this study by making time for small talk during the introduction to each of the three workshops. Further studies should research the role of psychological safety (38) in resilience and related interventions to improve resilient performance.

## 6. Conclusion

For interventions seeking to improve healthcare quality, objects prove to be valuable instruments for translating and transforming knowledge into practice and to promote reflection, motivation, the bridging of hierarchies and collaborative learning (3, 21). The novel resilience tool described here was found to serve as different forms of objects during the introduction of an unfamiliar resilience perspective to healthcare professionals. It provided a means to develop shared reflection, understanding, focus, and language for the different professional disciplines and settings involved. The resilience tool acted as a boundary object for the development of shared understanding and language, as an epistemic object for the development of shared focus, and as an activity object within the shared reflection sessions. Factors that enabled the internalization of the unfamiliar resilience perspective were to provide active facilitation of the workshops, give repeated explanations of unfamiliar concepts, provide relatedness to own context, and promote psychological safety in the workshops.

In sum, observations from the test phase of the RiH learning tool showed how these different objects were crucial in making tacit knowledge explicit which is key to improving healthcare quality and promote learning processes across healthcare services.

## Data availability statement

The raw data supporting the conclusions of this article will be made available by the authors, without undue reservation.

## Author contributions

HL, CH-D, and SW advanced the initial idea for the article. CH-D, VG, ER, HD, BF, HW, and HL contributed to the facilitation of workshops and in the data collection. HL drafted the manuscript with significant contributions from CH-D, VG, ER, HD, BF, HW, and SW. HL led the analysis process while all authors contributed to the analysis process at different stages. All authors have read and approved the final manuscript.

## Funding

This study was funded by the Norwegian research council, as a sub study under the overall Resilience in healthcare project (RiH) project number 275367. The Resilience in Healthcare Research Program has received funding from the Research Council of Norway from the FRIPRO TOPPFORSK program, grant agreement no. 275367. The University of Stavanger, Norway; NTNU Gjøvik, Norway; and The Norwegian Air Ambulance support the study with in-kind funding.

## Conflict of interest

The author SW is a member of the editorial board (Associate Editor).

The remaining authors declare that the research was conducted in the absence of any commercial or financial relationships that could be construed as a potential conflict of interest.

## Publisher’s note

All claims expressed in this article are solely those of the authors and do not necessarily represent those of their affiliated organizations, or those of the publisher, the editors and the reviewers. Any product that may be evaluated in this article, or claim that may be made by its manufacturer, is not guaranteed or endorsed by the publisher.
